# Early life adversity is associated with a smaller hippocampus in male but not female depressed in-patients: a case–control study

**DOI:** 10.1186/s12888-017-1233-2

**Published:** 2017-02-15

**Authors:** Romain Colle, Tomoyuki Segawa, Marie Chupin, Minh Ngoc Thien Kim Tran Dong, Patrick Hardy, Bruno Falissard, Olivier Colliot, Denis Ducreux, Emmanuelle Corruble

**Affiliations:** 1 INSERM UMRS 1178, Team “Depression and Antidepressants”, 94275 Le Kremlin Bicêtre, France; 2 Faculté de Médecine Paris Sud, Univ. Paris-Sud, 94275 Le Kremlin Bicêtre, France; 3 Service de Psychiatrie, Hôpital Bicêtre, Hôpitaux Universitaires Paris Sud, Assistance Publique-Hôpitaux de Paris, 94275 Le Kremlin Bicêtre, France; 4 INSERM U1127, F-75013 Paris, France; 5 CNRS, UMR 7225, 75013 Paris, France; 6 Sorbonne Universités, UPMC Univ. Paris 06, UMR S 1127, F-75013 Paris, France; 7 Institut du Cerveau et de la Moelle épinière, ICM, F-75013 Paris, France; 8 Inria, Aramis project-team, Centre de Recherche de Paris, Paris, France; 9 Departments of Neuroradiology and Neurology, AP-HP, Hôpital de la Pitié-Salpêtrière, F-75013 Paris, France; 10 CNRS IR4M, UMR 8081, 94275 Le Kremlin Bicêtre, France; 11 Service de Neuroradiologie, Hôpital Bicêtre, Hôpitaux Universitaires Paris Sud, Assistance Publique Hôpitaux de Paris, 94275 Le Kremlin Bicêtre, France; 12 INSERM, Université Paris-Saclay, Univ. Paris-Sud, UVSQ, CESP, Villejuif, France

**Keywords:** Early life adversity, Hippocampal volume, Sex, Major depressive disorder, Major depressive episode, MRI, Automated segmentation

## Abstract

**Background:**

Three studies assessed the association of early life adversity (ELA) and hippocampal volumes in depressed patients, of which one was negative and the two others did not control for several potential confounding variables. Since the association of ELA and hippocampal volumes differ in male and female healthy volunteers, we investigated the association of ELA and hippocampal volumes in depressed patients, while focusing specifically on sex and controlling for several relevant socio-demographic and clinical variables.

**Methods:**

Sixty-three depressed in-patients treated in a psychiatric setting, with a current Major Depressive Episode (MDE) and a Major Depressive Disorder (MDD) were included and assessed for ELA. Hippocampal volumes were measured with brain magnetic resonance imaging (MRI) and automatic segmentation. They were compared between patients with (*n* = 28) or without (*n* = 35) ELA. After bivariate analyses, multivariate regression analyses tested the interaction of sex and ELA on hippocampal volume and were adjusted for several potential confounding variables. The subgroups of men (*n* = 26) and women (*n* = 37) were assessed separately.

**Results:**

Patients with ELA had a smaller hippocampus than those without ELA (4.65 (±1.11) cm^3^ versus 5.25 (±1.01) cm^3^), bivariate: *p* = 0.03, multivariate: *HR* = 0.40, 95%CI [0.23;0.71], *p* = 0.002), independently from other factors. This association was found in men (4.43 (±1.22) versus 5.67 (±0.77) cm^3^), bivariate: *p* = 0.006, multivariate *HR* = 0.23, 95%CI [0.06;0.82], *p* = 0.03) but not in women.

**Conclusion:**

ELA is associated with a smaller hippocampus in male but not female depressed in-patients. The reasons for this association should be investigated in further studies.

## Background

Early Life Adversity (ELA) has deleterious consequences during childhood and throughout life [1]. ELA is found in one out of two adult patients with Major Depressive Disorder (MDD) [2–7]. And adult patients with MDD have a two-fold higher rate of ELA compared to healthy individuals [8].

Interestingly, ELA may have a different impact on men and women. Indeed, epidemiological data show that ELA increases the risk for depression differently in men and women [9]. Moreover, the brain maturation [10] and reorganization of the inhibitory control network [11] after ELA may differ between men and women.

Recently, biomarker research in MDD [12] has focused on the hippocampus. In particular, hippocampal volume has been identified as a promising biomarker, smaller in MDD patients as compared to healthy subjects [13, 14]. Moreover, functional Magnetic Resonance Imaging (fMRI) studies have shown changes in hippocampal activation associated with ELA (for review see Hart et al. 2012 [15]). In MDD, there are two studies showing differences in fMRI depending on the presence or absence of ELA. The first one in MDD children (20 boys and 22 girls) [16] reports that higher levels of ELA are associated with higher hippocampal activation in response to sad faces. The second one in 38 adult MDD patients reports that ELA is associated with a lower resting state connectivity in the limbic network, including the hippocampus [17]. In these two studies however, the impact of sex is not assessed.

Paradoxically, whereas the structural MRI biomarkers could be more easily transferable to clinical practice than functional MRI biomarkers, the literature linking ELA and hippocampal volumes in MDD is relatively poor, since only three studies are available [5, 18, 19]. The first study [18] performed in a sample of 31 women with remitted MDD fail to show an association between ELA and hippocampal volumes, even after controlling for age. In a second study of 37 MDD patients (16 men and 21 women) assessed with voxel based morphometry, Chaney et al. (2014) [5] report lower hippocampal grey matter in patients with ELA as compared to those without ELA, for the right but not for the left hippocampus. In a third study of 85 MDD patients (31 men and 54 women) assessed with a morphometric approach [19], Opel et al. (2014) show that ELA is associated with reduced hippocampal volumes. However, neither Chaney et al. (2014) nor Opel et al. (2014) controlled for socio-demographical or clinical variables, nor assessed specifically the role of sex. This point is crucial since hippocampal volumes are larger in men than in women [20, 21] and since a study in the general population shows that men with ELA have smaller hippocampal volumes than men without ELA whereas no difference is shown in women [22]. Regarding other relevant socio-demographic and clinical variables, older age [23], early age at onset of MDD [13], longer MDD duration [24], suicide attempts [25, 26] and smaller brain volume [20], are associated with smaller hippocampal volumes. Conversely, medication with antidepressants is associated with greater hippocampal volume [27]. And ELA is associated with early age at onset of MDD [28, 29] and higher depression severity [30].

Hence, the aim of our study was to investigate the association of ELA and hippocampal volumes in depressed patients, while focusing specifically on sex and controlling for several relevant socio-demographic and clinical variables.

## Methods

### Design

In this mono-centered study, the association between ELA and hippocampal volumes was assessed in adult depressed in-patients. This study was registered by the Commission Nationale de l’Informatique et des Libertés (CNIL) and was approved by the Ethics Committee of Paris-Boulogne, France, and conformed to international ethical standards and the latest version of the Declaration of Helsinki.

### Patients

Consecutive in-patients, aged 18–65 years, with a diagnosis of a current Major Depressive Episode (MDE) in a context of MDD (DSM-IVTR) based on the Mini International Neuropsychiatric Interview (MINI) [31] and a Hamilton Depression Rating Scale 17 items (HDRS) [32] score of 18 or more, are included, prior to beginning a new antidepressant treatment. Patients with bipolar disorders, psychotic disorders, organic brain syndromes, unstable medical conditions, and contra-indications to cerebral MRI are not included. Alcohol dependence and marijuana use were associated with smaller hippocampal volume [33, 34] and also with ELA [35, 36] and MDD [37, 38]. Accordingly, patients with current substance abuse or dependence (DSM-IVTR) were not included in this study. In order to have a representative sample and increase the generalizability of our results, anxiety disorders and personality disorders, which are frequently comorbid with MDD and with ELA, are not excluded. Written informed consent of the participants is obtained after the nature of the procedures had been fully explained. All patients are hospitalized in the department of psychiatry of Bicêtre university hospital. They are systematically assessed by a psychiatrist for sex, age, age at onset of MDD, history of suicide attempt, depression severity with HDRS and lifetime medication with antidepressants. 68 patients were included but five patients were excluded from the analysis because of poor quality of hippocampal segmentations and/or MRI artefacts leading to an unreliable estimation of hippocampal volume. The sample analyzed comprises 63 patients. Their mean age is 46.4 (±12.4) years, 37 (58.7%) are women, 7 (11.1%) have a low educational level (French elementary school level, age: 2–11 years), 31 (49.2%) have a middle educational level (French secondary school level, age: 11–18 years), 25 (39.7%) have a high educational level (French university level, age: 18 years and older), and 31 (49.2%) are married. Regarding their MDD, 44 (69.8%) have a recurrent MDD, the mean number of previous MDE is 2.4 (±1.6), the mean age at onset of MDD is 37.5 (±15.6) years, the mean MDD duration is 8.7 (±11.4) years, and 24 (38.1%) patients are lifetime suicide attempters. Regarding antidepressants, 49 (78.8%) were prescribed an antidepressant medication in the past and the mean duration of previous antidepressant treatment was 2.8 (±11.4) years. Thirty-four (54.0%) patients received an antidepressant medication the week before assessment (selective serotonin reuptake inhibitors: *n* = 8 (23.5%), serotonin and norepinephrin reuptake inhibitors: *n* = 15 (44.1%), tricyclics: *n* = 5 (14.7%), others: *n* = 6 (17.6%)), but this medication regimen was stopped two days before the assessment.

### Early life adversity

ELA is defined here by either the death of caregivers or child abuse/maltreatment [1, 39]. Child abuse/maltreatment was defined by all forms of physical and/or emotional ill-treatment, sexual abuse, neglect or negligent treatment or commercial or other exploitation, resulting in actual or potential harm to the child’s health, survival, development or dignity in the context of a relationship of responsibility, trust or power [40].

Assessment of ELA was performed by 2 independent psychiatrists (TS and RC), using patient health records. TS and RC were not involved in the treatment of these patients and were blind to hippocampal volumes. Patient health records were documented during the hospitalization. Informations were obtained from the patients and close relationships, by several professionals, including senior psychiatrists, resident psychiatrists, general practitioners, other physicians who took care of this particular patient, nurses, psychologists and social workers. Indeed, during their hospitalization, patients benefited from multiple interviews with these professionals, who were blind from the objectives of the study. After the end of the hospitalization, each patient’s health record was screened for the presence/absence of each component of ELA. ELA, i.e. death of caregiver, sexual abuse, emotional abuse, physical and emotional neglect, verbal abuse [39, 41, 42], was assessed as present if there was at least the death of a caregiver or one type of abuse (physical, verbal, sexual or emotional) or neglect (physical or emotional). Otherwise, ELA was assessed as absent. Afterwards, TS and RC reviewed the health records during a consensus group meeting supervised by EC, in order to obtain an agreement on the presence/absence of ELA.

### Brain magnetic resonance imaging

Brain MRI acquisition methods were previously described [25]. Brain MRI acquisitions were performed on 1.5 T (*n* = 47) or 3 T (*n* = 16) Philips systems. All subjects were scanned with a routine whole brain T1-weighted 3D sequence. These images were acquired with a resolution of either: sequence 1: 0.6 × 0.6 × 0.7 (interpolated) in sagittal plane (*n* = 10); sequence 2: 0.94 × 0.94 × 1.00 in axial plane (*n* = 37); sequence 3: 0.88 × 0.88 × 1.1 in sagittal plane (*n* = 16). Since there were three different MRI acquisition sequences, the MRI acquisition method was added as a covariable in the multivariate analyses. The different acquisition sequences were not statistically different in men and women and in patients with or without ELA. The segmentation of the hippocampus was performed using the fully automatic SACHA software [43–46]. This approach segments the hippocampus based on competitive region-growing between the hippocampus and amygdala. It includes prior knowledge of the location of the hippocampus and the amygdala derived from a probabilistic atlas and on the relative positions of these structures with respect to anatomical landmarks, which are automatically identified. All resulting segmentations were assessed for segmentation quality (from 0 for worst quality to 4 for perfect quality) by trained raters (R.C and M.C), blind to the sociodemographic and clinical data. Only high quality segmentations (quality score ≥ 2) were included in the analyses. Three variables of interest were studied on the basis of previous published papers [14, 47, 48]. Total hippocampal volume was the main assessment criterion. Right and left hippocampal volumes were also analyzed individually. Total brain volumes were estimated with SPM5 to control hippocampal volumes for this variable.

### Statistical methods

ELA is the main independent variable and total hippocampal volume is the main dependent variable. After descriptive analyses, bivariate analyses were performed using Chi2 tests for categorical variables and Wilcoxon tests for continuous variables. After bivariate analyses, multivariate linear regressions were computed testing the interaction of sex and ELA on hippocampal volume and were adjusted on several variables, which could be confounders of the association between ELA and the hippocampal volumes. This possibility is based on data from the literature as well as results from bivariate analyses. Accordingly, the analyses were adjusted on age, brain volumes, MRI acquisition method, age at onset of MDD, history of suicide attempts, previous antidepressant medication and HDRS scores. Longer MDD duration was previously reported to be associated with smaller hippocampal volume [24]. However, since this variable was negatively correlated with age at onset of MDD (*r* = −0.6, *p* = 0.000002 in our sample), only age at onset of MDD was included in the multivariate analysis. Moreover, since the use of 3 different MRI acquisition sequences may bias our results, the multivariate analyses were adjusted on the MRI acquisition method (sequence 1, sequence 2 and sequence 3).

In addition, the interaction between ELA and sex was specifically studied in the multivariate linear regressions. In the case of significant interactions, bivariate analyses were performed to test the association between ELA and hippocampal volumes in the subgroups of women and men.

All tests were two-tailed. Significance level was defined as *p* < 0.05. The software used was R 3.2.2 (www.r-project.org).

## Results

ELA is found in 28 (44.4%) patients. Multiple ELA are found in 12 patients. The types of ELA are: death of caregiver (11 (17.4%)), physical abuse (8 (12.7%)), emotional abuse (4 (6.3%)), sexual abuse (2 (3.2%)), verbal abuse (5 (7.9%)), physical neglect (4 (6.3%)) and emotional neglect (17 (27.0%)).

Patients with and without ELA do not differ in terms of sex and other socio-demographic variables and MDD features, except for the age at onset of MDD, which is earlier in patients with ELA as compared to those without ELA (Table 1).

**Table 1:** Socio-demographical and clinical data in patients with or without early life adversity

	ELA+ (*n* = 28)	ELA- (*n* = 35)
Women n (%)	19 (67.9)	18 (51.4)
Age (years) (m (sd))	43.6 (12.4)	48.5 (12.1)
Educational level (%)		
Low	2 (7.1)	5 (14.3)
Middle	17 (60.7)	14 (40.0)
High	9 (32.1)	16 (45.7)
Age at onset of MDD (m (sd)) ^a^	32.9 (15.1)	41.2 (15.3)
MDD duration (m (sd))	10.7 (12.5)	7.1 (10.3)
Recurrent MDD (%)	21 (75.0)	23 (65.7)
Number of previous MDE (m (sd))	2.3 (1.6)	2.4 (1.7)
HDRS (m (sd))	23.8 (5.2)	24.5 (5.7)
Previous antidepressant medication (%)	22 (78.6)	27 (77.1)
Suicide Attempts (%)	10 (35.7)	14 (40.0)

*ELA* early life adversity, *ELA*+ presence of early life adversity, *ELA*- absence of early life adversity, *MDD* major depressive disorder, *MDE* major depressive episode, *HDRS* hamilton depression rating scale 17 items, ^a^: *w* = 619, *p* = 0.04

**Fig. 1:**
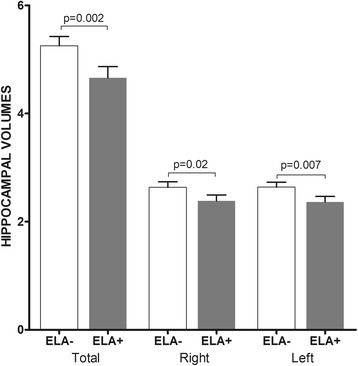
Early life adversity and hippocampal volumes. Hippocampal volumes: m (sem) (cm3); ELA+: Patients with early life adversity; ELA-: Patients without early life adversity; p: *p* value controlled for sex, age, brain volumes, MRI acquisition method, age at onset of MDD, history of suicide attempts, previous antidepressant medication and HDRS

Significant and independent associations are shown between ELA and hippocampal volumes (Table 2). As compared to patients without ELA, patients with ELA have smaller total hippocampal volumes (Fig. 1), as evidenced in the bivariate (*w* = 644.5, *p* = 0.03) and multivariate (*HR* = 0.40, 95%CI [0.23; 0.71], *p* = 0.002) analyses. They also had smaller right hippocampal volumes (bivariate: *w* = 570, *p* = 0.10; multivariate: *HR* = 0.67, CI95%CI [0.49;0.93], *p* = 0.02) and smaller left hippocampal volumes (bivariate: *w* = 574, *p* = 0.06; multivariate: *HR* = 0.66, 95%CI [0.48;0.89], *p* = 0.007).

**Table 2:** Results of multivariate models for total, right and left hippocampal volumes

	Total Hippocampal Volume		Right Hippocampal Volume		Left Hippocampal Volume	
	estimate	*p*	estimate	*p*	estimate	*p*
ELA+ (*vs* ELA-)	−0.91	**0.002**	−0.40	**0.02**	−0.43	**0.007**
Sex (women *vs* men)	0.37	0.26	0.18	0.31	0.18	0.31
Age	0.00	0.81	0.00	0.16	0.00	0.83
Brain volume	0.00	0.07	0.00	0.52	0.00	**0.004**
MRI acquisition method						
(sequence 2 *vs* 1)	1.28	**0.002**	0.85	**0.0003**	0.37	0.08
(sequence 3 *vs* 1)	1.31	**0.003**	0.77	**0.002**	0.59	**0.01**
MDD age at onset	−0.01	0.68	−0.00	0.66	−0.00	0.76
History of suicide attempts (yes *vs* no)	−0.66	**0.02**	−0.37	**0.01**	−0.17	0.25
Previous antidepressant medication (yes *vs* no)	−0.01	0.98	−1.17	0.25	0.18	0.36
HDRS score	−0.01	0.61	−0.01	0.58	−0.01	0.49
ELA and Sex interaction	1.16	**0.03**	0.52	0.10	0.61	**0.03**

*ELA* early life adversity, *ELA*+ presence of early life adversity, *ELA*- absence of early life adversity, MRI acquisition: sequence 1: 0.6 × 0.6 × 0.7 (interpolated) in sagittal plane, sequence 2: 0.94 × 0.94 × 1.00 in axial plane; sequence 3: 0.88 × 0.88 × 1.1 in sagittal plane, *MDD* major depressive disorder, *HDRS* hamilton depression rating scale 17 items; bold: *p* value < 0.05

In multivariate analyses (Table 2), significant and independent associations are also shown between three other variables (brain volume, MRI acquisition method and history of suicide attempts) and hippocampal volumes.

A significant interaction between ELA and sex is shown for total hippocampal volumes (*p* = 0.04) and left hippocampal volumes (*p* = 0.03) (Table 2).

In the subgroup of men (*n* = 26), those with ELA have smaller total, right and left hippocampal volumes than those without ELA (respectively *p* = 0.06, *p* = 0.01 and *p* = 0.02 in bivariate analyses, Table 3). Multivariate analyses adjusted for age, brain volumes, MRI acquisition method, age at onset of MDD, history of suicide attempts, previous antidepressant medication and HDRS show that men with ELA have smaller total and right hippocampal volumes, but not left hippocampal volumes, than men without ELA (respectively *HR* = 0.23, 95%CI [0.06; 0.82], *p* = 0.03; *HR* = 0.52, 95%CI [0.27; 1.00], *p* = 0.05; and *HR* = 0.72, 95%CI [0.23; 2.25], *p* = 0.55).

**Table 3:** Early life adversity and hippocampal volumes in men and women

	Women			Men		
	ELA + (*n* = 19)	ELA−(*n* = 18)	*p*	ELA + (*n* = 9)	ELA−(*n* = 17)	*p*
Total hippocampal volume (cm^3^) (m (sd))	4.77 (1.07)	4.85 (1.07)	0.99	4.43 (1.22)	5.67 (0.77)	**0.006**
Right hippocampal volume (cm^3^) (m (sd))	2.43 (0.59)	2.45 (0.64)	0.99	2.28 (0.57)	2.82 (0.49)	**0.01**
Left hippocampal Volume (cm^3^) (m (sd))	2.41 (0.50)	2.43 (0.51)	0.98	2.23 (0.66)	2.86 (0.45)	**0.02**

*ELA* early life adversity, *ELA*+ presence of early life adversity, *ELA* − absence of early life adversity, *p p* values in bivariate analyses, **bold:** *p* value < 0.05

**Fig. 2:**
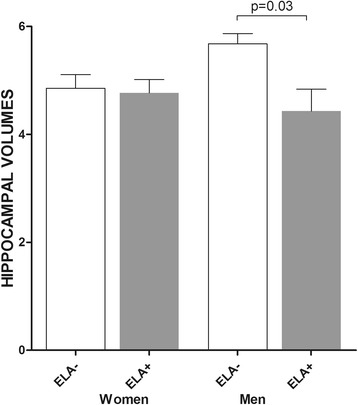
Early life adversity and total hippocampal volumes in men and women. Hippocampal volumes: m (sem) (cm3); ELA+: Patients with early life adversity; ELA−: Patients without early life adversity; p: *p* value adjusted for age, brain volumes, MRI acquisition method, age at onset of MDD, history of suicide attempts, previous antidepressant medication and HDRS score

In the subgroup of women (*n* = 37), no difference of hippocampal volumes (total, right or left) is shown between those with and without ELA (Table 3, Fig. 2).

## Discussion

Our results show that ELA is associated with a smaller hippocampus in male but not female depressed in-patients treated in a psychiatric setting, even after controlling for several relevant socio-demographic and clinical variables. The main strengths of this study are that it focuses specifically on the effect of sex and that it controls for several relevant variables, such as age, age at onset of MDD, history of suicide attempts, previous antidepressant medication and Hamilton Depression Rating Scale scores.

This result is coherent with the result of Samplin et al. (2013) in healthy volunteers [22], which shows that healthy men with ELA have smaller hippocampal volumes than healthy men without ELA, while no difference was evidenced in women. It suggests that our result might not be specific to MDD patients. However, the effect could be more pronounced in depressed patients, since depressed men with ELA in our sample have a 21.9% lower hippocampal volume than depressed men without ELA, whereas the difference was 7.3% in the sample of male healthy volunteers of Samplin et al. (2013) [22]. Other studies concerning healthy subjects [19, 49–51] report smaller hippocampal volumes in individuals with ELA. However, the effect of gender was not specifically assessed in these studies. Our results are also coherent with those of Lenze et al. (2008) [18], who show no association between ELA and hippocampal volumes in a sample of 31 women with MDD. But our results go beyond those of Chaney et al. (2014) and Opel et al. (2014), who did not control for socio-demographical or clinical variables, and did not assess specifically the role of sex. Indeed, our study is the first one showing a specific effect of sex on the association between ELA and hippocampal volumes in MDD, this association being evidenced only in men. Moreover, our results are in line with those of the two fMRI studies concerning MDD [16, 17], although these two studies did not assess the effect of sex. Moreover, our results are in line with those of a study in patients with chronic psychosis [52] although the specific effect of sex was not assessed. In addition, we show a specific and independent effect of the history of suicide attempts, which is coherent with previous results [25, 26]. The non-inclusion of patients with current substance abuse or dependence is one of the strengths of this study. Thus alcohol consumption and marijuana use cannot bias our results.

It should be highlighted that several points argue for the generalizability of our results. They are in line with those of the literature for the frequency of ELA (44.4% in our sample, 54% in Chaney et al. (2014) [5], 51.4% in Gerritsen et al. (2015) [6], for the magnitude of hippocampal volumes [46, 53] and for the association between ELA and earlier age at onset of MDD [28, 29].

Nevertheless, our study has some limitations. Firstly, ELA is retrospectively assessed as a dichotomous variable (absent/present), leading to possible memory biases among patients. However, we used a clinical assessment method, which could catch more relevant events than self-assessment methods. Our method is based on medical records, several information sources (i.e. patients and close relationships) and multiple interviews with multiple interviewers leading to possible higher sensitivity to detect ELA than a one-time self-assessment subjected to memory biases. However, our method is difficult to use with out-patients or healthy subjects, since it is time consuming, but it may be particularly relevant for in-patients. Nonetheless, our results should be replicated with validated assessment methods of ELA. This may include a clinician administered interview Childhood Experience of Care and Abuse (CECA) This may include a clinician administered interview of the Childhood Experience of Care and Abuse (CECA), or its self-report version, the CECA questionnaire, which assess several dimensions such as lack of parental care (neglect and antipathy), parental physical abuse, and sexual abuse from any adult before age 17 [54], or the Childhood Trauma Questionnaire [41] which is also a self-report method and the most used method assessing emotional and physical neglect and emotional, physical, and sexual abuse. However, standardized methods in depressed patients lead to frequencies of ELA which are similar to our results (44.4% in our sample, 54% in Chaney et al. (2014) [5] with the Childhood Trauma Questionnaire [41], 51.4% in Gerritsen et al. (2015) [6] and with the Nemesis Trauma Interview [55].

Secondly, due to the small sample size, especially in the subgroup of men, we cannot exclude that our results may be false positives. However, their coherence with those shown in healthy volunteers [22] argue for their validity.

Thirdly, the power of the study was not sufficient to analyze the impact of specific types of ELA. Fourthly, the impact of anxiety disorders or personality disorders was not analyzed because these variables were not specifically assessed here.

Fifthly, this sample comprises only MDD in-patients treated in a psychiatric setting. Thus, our results cannot be generalized to the whole population of MDD patients. And, lastly, these results cannot be interpreted in terms of causality.

Nonetheless, the explanation for this association is unclear. However, it could be suggested that there is a mediating role of the dysregulation of the hypothalamic pituitary adrenal (HPA) axis Indeed, ELA is associated with a dysregulation of the HPA axis (for review see [56] and Struber et al. 2014 [57]). In addition, cortisol can have detrimental effects on hippocampal neurons [58]. Interestingly, a sex-dependent impact of ELA on saliva daily cortisol levels has been reported. Boys with ELA have a higher daily cortisol level than girls with ELA [59]. This higher cortisol level may explain the lower hippocampal volume shown in men but not in women with major depression. However, further studies are needed to test this hypothesis.

If they could be replicated in further studies, our results may have several implications. Generally speaking, it would be useful to pay more attention to ELA in boys and men, their assessment and potential impact on hippocampal volumes and MDD. More specifically, the benefits of psychotherapy in boys and men with ELA should be assessed to a greater degree. Finally, since men receive less appropriate mental health care than women for MDD [60], it would be useful to improve care access, specifically for men in order to treat MDD earlier in men. Finally, our results and their potential explanations should be studied further in longitudinal studies assessing ELA in children, and especially boys, and following them prospectively for both hippocampal volumes and MDD.

## Conclusion

Early life adversity is associated with a smaller hippocampus in male but not female depressed in-patients, even after controlling for several potential confounders. The reasons for this association should be investigated in further studies.

## Abbreviations

CNIL: Commission Nationale de l’Informatique et des Libertés (CNIL)
ELA: Early Life Adversity
HDRS: Hamilton Depression Rating Scale
MDD: Major Depressive Disorder
MDE: Major Depressive Episode
MRI: Magnetic Resonance Imaging
